# Supplementary material for: Comparison of local large language models for extraction of signs and symptoms data from electronic health records
Source: PLoS One. 2026 Jun 10;21(6):e0350625. doi: 10.1371/journal.pone.0350625 (PMC13252767; doi:10.1371/journal.pone.0350625)
Supplement: S1 Appendix — (PDF) [file pone.0350625.s001.pdf]

Appendix

Comparison of local large language models for extraction of signs and symptoms data from electronic health records. I. Spiero, M.H. Rijk, M.A. Scheeres, F.H. Rutten, G.J. Geersing, T.N. Platteel, K.G.M. Moons, L. Hooft, J.A.A. Damen, R.P. Venekamp, A.M. Leeuwenberg (2025).

A. SELECTION OF LARGE LANGUAGE MODELS

Selection criteria

We defined a set of selection criteria that indicate the suitability of a large language model (LLM) for the purpose of our study which targets the extraction of data from Dutch electronic health records (EHRs):

- *Local applicability*: Since clinical notes and EHRs in general contain a multitude of privacy sensitive data, it is mandatory that the model is able to be completely implemented on local hardware, as no EHR data is allowed to be transmitted from the environment it is stored on. For this reason, only open-source, locally applicable LLMs could be selected.
- *Availability of a pre-trained model*: The model must be pre-trained, as we do not have the computational power and amount of data to train a model from scratch. We do however want to be able to further fine-tune our models on our own data and convert them into prompt-based classifiers as well.
- *Parameter count of a model*: The number of parameters in a model represents the size and complexity of a model and thus correlates with the computational power required to run it. As computational power is a limiting factor due to local application of the model, we needed to balance between having enough parameters for sufficient LLM performance and the ability to run the LLM run on a virtual desktop.
- *Dutch language competency*: It is vital for the model to be able to handle Dutch language data, either through optimization for the Dutch language or being completely trained on a Dutch dataset.
- *Medical text familiarity*: Models trained or fine-tuned on medical texts are more likely to be able to correctly process our domain-specific knowledge and jargon and are thus preferred.

Model search

To retrieve a list of relevant models, a literature search was performed and combined with a direct search for models on the Hugging Face website. These websites provide comprehensive filtering options, enabling us to find models that can be run offline rather easily. To find the models via HuggingFace, the following filters were applied during a search performed on 18 October 2023:

- *Tasks*: Text Classification, Zero-Shot Classification, Conversational, Text Generation, Fill-Mask
- *Libraries*: PyTorch, TensorFlow, Transformers, Sentence Transformers, Adapter Transformers,
- *Datasets*: *Not specified*
- *Languages*: Dutch
- *Licences*: *Not specified*
- *Other*: *Not specified*

In the context of this research, we focused our search of models on LLMs that show promise in classification tasks. The final list of potential models for those tasks and their relevant features is presented in Table A1.

Table A1 | The large language models that were retrieved by our search including the characteristics on suitability for extraction of information from Dutch electronic health records.

Model	Base Model	Number of parameters	Prompt-based model available	Local	Pretrained	Dutch	Medical	Open-source	Relevant paper	Model link	Suitable
BERTje	BERT	109M	No	Yes	Yes	Yes	No	Yes	Link	GH | HF	Yes
GPT-2 (recycled for Dutch)	GPT-2	129M or 369M	No	Yes	Yes	Yes	No	Yes	Link	HF small, medium	Yes
GPT-2 XL	GPT-2	1.5B	No	Yes	Yes	Multilingual	No	Yes	Link	HF	Yes
DistilBERT-nl	BERT	69M	No	Yes	Yes	Yes	No	Yes	Link	HF	Yes
GPT-3.5/4	GPT-3.5/4	154B / 1.76T	Yes	No	Yes	Multilingual	No	No	Link3.5 / Link4	NA	No
Legal BERT	BERT	295M	No	Yes	Yes	Yes	No	Yes	Link	HF	No
medRoBERTa.nl	BERT	117M	Yes	Yes	No (possibly via contact)	Yes	Yes	Yes	Link	GH	Yes
Google Bard	Lambda	137B	Yes	No	Yes	Multilingual	No	No	NA	NA	No
LLaMa	LLaMa	65B	Yes	Yes	Yes	Multilingual	No	No	Link	HF	Yes
LLaMa 2	LLaMa2	7B to 70B	Yes	Yes	Yes	Multilingual	No	No	Link	HF	Yes
ChatDoctor	LLaMa-7B	7B	Yes	Yes	No	Multilingual	Yes	No	Link	GH	Yes
RoBERTa	BERT	355M	Yes	Yes	Yes	Multilingual	No	Yes	Link	HF	Yes
RobBERT-2023	BERT	117M	Yes	Yes	Yes	Yes	No	Yes	Link	HF	Yes
DialoGPT	None	117M, 345M or 762M	Yes	Yes	Yes	Multilingual	No	Yes	Link	GH	Yes, but not used

Final selection

After our search for available models, two models were selected as most suitable for our task (see computation details of each model in Table A2):

- *MedRoBERTa.nl* [1]
  - Description: A Dutch medical model created by researchers at Vrije Universiteit Amsterdam, who used 13GB of text data from Dutch hospital notes to train an altered version of the RoBERTa model. This model has shown to perform better on this data in odd-one-out tasks when compared to general Dutch LLMs and is thus likely to perform well in our research.
  - Motivation: Of all chosen models, MedRoBERTa.nl is the only one directly trained on Dutch medical data. Consequently, this model is expected to perform very well on the dataset. One limitation is that the parameter count of the model is rather low by modern standards, which, while being a positive factor in terms of computational efficiency, might reduce its performance.
- *RobBERT* [2]
  - Description: RobBERT is a model built upon the foundation of BERT, serving as the core architecture. BERT was released as a multilingual model, and RobBERT was fine-tuned as a language-specific model, as research points out that doing so generally results in higher performance.
  - Motivation: RobBERT's strong performance on various NLP tasks and proven ability to handle Dutch text make it a suitable candidate for our investigation.

Table A2 | The number of parameters of the selected MedRoBERTa.nl and RobBERT models. Both models were implemented as direct classifier and as prompt-based classifier. Prompt-based classifiers had a larger number of parameters compared to the direct classifiers due to extra layers being added by the EncoderDecoder model (with the base multilingual BERT model as decoder).

Classifier	Model	Number of parameters
**Direct**	*MedRoBERTa.nl*	125,980,419
	RobBERT	116,764,419
**Prompt-based**	*MedRoBERTa.nl*	263,859,258
	RobBERT	254,643,258

[1] Verkijk, S., & Vossen, P. (2021). MedRoBERTa. nl: a language model for Dutch electronic health records. In *Computational Linguistics in the Netherlands* (Vol. 11, pp. 141-159).
[2] Delobelle, P., Winters, T., & Berendt, B. (2020). Robbert: a dutch roberta-based language model. *arXiv preprint arXiv:2001.06286*.

B. Performance metrics of the LLMs in extracting signs and symptoms from Dutch EHRs

Table A3 | The performance of the MedRoBERTa.nl and RobBERT models as direct classifier in terms of recall, precision, and F1-score for different training sample sizes. The models were trained to extract a total of nine signs and symptoms related to lower respiratory tract infection from Dutch primary care electronic health records.

						Per class metrics								
			Macro-averaged metrics			'Present'			'Absent'			'Not reported'		
**Model**	**Sign or symptom**	**Number of samples**	**Precision**	**Recall**	**F1-score**	**Precision**	**Recall**	**F1-score**	**Precision**	**Recall**	**F1-score**	**Precision**	**Recall**	**F1-score**
MedRoBERTa.nl	Chest pain	200	0,28	0,33	0,31	0,00	0,00	0,00	0,00	0,00	0,00	0,85	1,00	0,92
MedRoBERTa.nl	Chest pain	400	0,70	0,43	0,45	0,58	0,10	0,16	0,65	0,19	0,27	0,87	0,99	0,93
MedRoBERTa.nl	Chest pain	600	0,77	0,74	0,74	0,67	0,65	0,65	0,70	0,63	0,63	0,95	0,95	0,95
MedRoBERTa.nl	Chest pain	800	0,79	0,81	0,78	0,74	0,68	0,68	0,68	0,80	0,72	0,96	0,95	0,95
MedRoBERTa.nl	Chest pain	1000	0,80	0,82	0,80	0,69	0,76	0,72	0,74	0,74	0,73	0,96	0,95	0,96
MedRoBERTa.nl	Chest pain	1200	0,82	0,78	0,79	0,70	0,68	0,68	0,80	0,71	0,74	0,96	0,96	0,96
MedRoBERTa.nl	Chest pain	1400	0,83	0,78	0,80	0,68	0,72	0,69	0,84	0,67	0,74	0,96	0,96	0,96
MedRoBERTa.nl	Chest pain	1600	0,84	0,74	0,78	0,75	0,59	0,65	0,82	0,67	0,73	0,94	0,97	0,96
MedRoBERTa.nl	Chills	200	0,32	0,33	0,32	0,00	0,00	0,00	0,00	0,00	0,00	0,95	1,00	0,97
MedRoBERTa.nl	Chills	400	0,32	0,33	0,32	0,00	0,00	0,00	0,00	0,00	0,00	0,95	1,00	0,97
MedRoBERTa.nl	Chills	600	0,32	0,33	0,32	0,00	0,00	0,00	0,00	0,00	0,00	0,95	1,00	0,97
MedRoBERTa.nl	Chills	800	0,64	0,62	0,63	0,91	0,86	0,88	0,00	0,00	0,00	0,99	1,00	1,00
MedRoBERTa.nl	Chills	1000	0,62	0,62	0,62	0,88	0,85	0,86	0,00	0,00	0,00	0,99	1,00	0,99
MedRoBERTa.nl	Chills	1200	0,63	0,63	0,63	0,90	0,90	0,90	0,00	0,00	0,00	0,99	1,00	1,00
MedRoBERTa.nl	Chills	1400	0,64	0,61	0,62	0,92	0,82	0,85	0,00	0,00	0,00	0,99	1,00	1,00
MedRoBERTa.nl	Chills	1600	0,63	0,63	0,63	0,91	0,89	0,89	0,00	0,00	0,00	0,99	1,00	1,00
MedRoBERTa.nl	Confusion	200	0,32	0,33	0,33	0,00	0,00	0,00	0,00	0,00	0,00	0,97	1,00	0,98
MedRoBERTa.nl	Confusion	400	0,32	0,33	0,33	0,00	0,00	0,00	0,00	0,00	0,00	0,97	1,00	0,98
MedRoBERTa.nl	Confusion	600	0,32	0,33	0,33	0,00	0,00	0,00	0,00	0,00	0,00	0,97	1,00	0,98
MedRoBERTa.nl	Confusion	800	0,42	0,41	0,41	0,20	0,20	0,20	0,08	0,04	0,05	0,97	1,00	0,99
MedRoBERTa.nl	Confusion	1000	0,48	0,46	0,44	0,07	0,20	0,10	0,40	0,19	0,25	0,98	1,00	0,99
MedRoBERTa.nl	Confusion	1200	0,52	0,54	0,52	0,10	0,20	0,13	0,49	0,44	0,44	0,98	0,99	0,99

						Per class metrics								
			Macro-averaged metrics			‘Present’			‘Absent’			‘Not reported’		
**Model**	**Sign or symptom**	**Number of samples**	**Precision**	**Recall**	**F1-score**	**Precision**	**Recall**	**F1-score**	**Precision**	**Recall**	**F1-score**	**Precision**	**Recall**	**F1-score**
MedRoBERTa.nl	Confusion	1400	0,64	0,61	0,57	0,45	0,33	0,27	0,48	0,52	0,45	0,98	0,99	0,99
MedRoBERTa.nl	Confusion	1600	0,61	0,54	0,56	0,40	0,33	0,35	0,46	0,29	0,34	0,98	0,99	0,99
MedRoBERTa.nl	Cough	200	0,25	0,33	0,29	0,76	1,00	0,86	0,00	0,00	0,00	0,00	0,00	0,00
MedRoBERTa.nl	Cough	400	0,55	0,39	0,40	0,79	0,99	0,88	0,00	0,00	0,00	0,85	0,19	0,31
MedRoBERTa.nl	Cough	600	0,59	0,59	0,59	0,93	0,96	0,95	0,00	0,00	0,00	0,85	0,81	0,83
MedRoBERTa.nl	Cough	800	0,60	0,59	0,59	0,93	0,96	0,94	0,00	0,00	0,00	0,87	0,79	0,82
MedRoBERTa.nl	Cough	1000	0,60	0,60	0,60	0,94	0,96	0,95	0,00	0,00	0,00	0,87	0,85	0,86
MedRoBERTa.nl	Cough	1200	0,60	0,59	0,59	0,93	0,97	0,95	0,00	0,00	0,00	0,88	0,79	0,83
MedRoBERTa.nl	Cough	1400	0,60	0,60	0,60	0,94	0,96	0,95	0,00	0,00	0,00	0,87	0,85	0,86
MedRoBERTa.nl	Cough	1600	0,60	0,59	0,59	0,93	0,97	0,95	0,00	0,00	0,00	0,88	0,79	0,83
MedRoBERTa.nl	Crackles upon auscultation	200	0,55	0,49	0,47	0,00	0,00	0,00	0,66	1,00	0,80	0,97	0,47	0,63
MedRoBERTa.nl	Crackles upon auscultation	400	0,83	0,57	0,57	0,87	0,13	0,20	0,70	0,98	0,81	0,93	0,59	0,72
MedRoBERTa.nl	Crackles upon auscultation	600	0,88	0,77	0,81	0,81	0,77	0,79	0,85	0,94	0,89	0,97	0,61	0,74
MedRoBERTa.nl	Crackles upon auscultation	800	0,88	0,82	0,85	0,84	0,81	0,83	0,88	0,95	0,91	0,92	0,70	0,79
MedRoBERTa.nl	Crackles upon auscultation	1000	0,89	0,83	0,85	0,88	0,80	0,84	0,88	0,96	0,92	0,92	0,72	0,80
MedRoBERTa.nl	Crackles upon auscultation	1200	0,87	0,85	0,86	0,84	0,86	0,85	0,91	0,92	0,92	0,87	0,76	0,80
MedRoBERTa.nl	Crackles upon auscultation	1400	0,87	0,86	0,86	0,85	0,86	0,85	0,91	0,92	0,92	0,85	0,78	0,81
MedRoBERTa.nl	Crackles upon auscultation	1600	0,88	0,86	0,87	0,86	0,88	0,87	0,92	0,93	0,93	0,87	0,77	0,82
MedRoBERTa.nl	Fever	200	0,48	0,37	0,29	0,54	0,03	0,06	0,46	0,20	0,23	0,45	0,88	0,59
MedRoBERTa.nl	Fever	400	0,60	0,52	0,51	0,67	0,24	0,35	0,45	0,70	0,54	0,69	0,64	0,66
MedRoBERTa.nl	Fever	600	0,70	0,70	0,70	0,69	0,74	0,71	0,62	0,60	0,61	0,79	0,77	0,78
MedRoBERTa.nl	Fever	800	0,73	0,73	0,72	0,68	0,79	0,73	0,74	0,54	0,62	0,77	0,85	0,81
MedRoBERTa.nl	Fever	1000	0,73	0,73	0,72	0,70	0,75	0,72	0,69	0,59	0,64	0,79	0,84	0,81
MedRoBERTa.nl	Fever	1200	0,71	0,70	0,70	0,70	0,75	0,72	0,62	0,62	0,62	0,80	0,73	0,76
MedRoBERTa.nl	Fever	1400	0,72	0,72	0,72	0,70	0,81	0,74	0,67	0,61	0,64	0,80	0,75	0,77
MedRoBERTa.nl	Fever	1600	0,71	0,72	0,71	0,66	0,81	0,72	0,67	0,60	0,63	0,79	0,75	0,77
MedRoBERTa.nl	Ill appearance	200	0,23	0,33	0,27	0,00	0,00	0,00	0,00	0,00	0,00	0,69	1,00	0,82

						Per class metrics								
			Macro-averaged metrics			‘Present’			‘Absent’			‘Not reported’		
Model	Sign or symptom	Number of samples	Precision	Recall	F1-score	Precision	Recall	F1-score	Precision	Recall	F1-score	Precision	Recall	F1-score
MedRoBERTa.nl	Ill appearance	400	0,58	0,62	0,58	0,11	0,02	0,03	0,72	0,86	0,78	0,89	0,98	0,93
MedRoBERTa.nl	Ill appearance	600	0,86	0,83	0,84	0,75	0,64	0,69	0,91	0,88	0,90	0,93	0,96	0,94
MedRoBERTa.nl	Ill appearance	800	0,87	0,81	0,83	0,78	0,57	0,64	0,91	0,89	0,90	0,92	0,96	0,94
MedRoBERTa.nl	Ill appearance	1000	0,87	0,85	0,86	0,74	0,69	0,71	0,93	0,89	0,91	0,93	0,95	0,94
MedRoBERTa.nl	Ill appearance	1200	0,88	0,85	0,86	0,77	0,69	0,73	0,94	0,89	0,91	0,93	0,96	0,95
MedRoBERTa.nl	Ill appearance	1400	0,90	0,85	0,87	0,81	0,70	0,75	0,94	0,89	0,92	0,94	0,97	0,95
MedRoBERTa.nl	Ill appearance	1600	0,90	0,85	0,87	0,83	0,67	0,73	0,95	0,90	0,92	0,93	0,97	0,95
MedRoBERTa.nl	Shortness of breath	200	0,38	0,40	0,33	0,45	0,86	0,58	0,00	0,00	0,00	0,71	0,33	0,42
MedRoBERTa.nl	Shortness of breath	400	0,43	0,48	0,44	0,53	0,84	0,65	0,00	0,00	0,00	0,77	0,60	0,66
MedRoBERTa.nl	Shortness of breath	600	0,80	0,69	0,69	0,79	0,89	0,84	0,75	0,27	0,36	0,86	0,91	0,88
MedRoBERTa.nl	Shortness of breath	800	0,85	0,80	0,81	0,86	0,91	0,88	0,78	0,59	0,66	0,90	0,91	0,90
MedRoBERTa.nl	Shortness of breath	1000	0,85	0,82	0,83	0,86	0,93	0,89	0,78	0,65	0,71	0,92	0,89	0,90
MedRoBERTa.nl	Shortness of breath	1200	0,86	0,82	0,83	0,89	0,92	0,90	0,77	0,62	0,68	0,91	0,93	0,92
MedRoBERTa.nl	Shortness of breath	1400	0,88	0,83	0,85	0,87	0,93	0,90	0,85	0,64	0,73	0,91	0,91	0,91
MedRoBERTa.nl	Shortness of breath	1600	0,85	0,82	0,83	0,87	0,93	0,90	0,75	0,65	0,69	0,92	0,90	0,91
MedRoBERTa.nl	Sputum	200	0,24	0,33	0,28	0,00	0,00	0,00	0,00	0,00	0,00	0,73	1,00	0,85
MedRoBERTa.nl	Sputum	400	0,50	0,37	0,36	0,74	0,15	0,21	0,00	0,00	0,00	0,76	0,98	0,85
MedRoBERTa.nl	Sputum	600	0,58	0,60	0,59	0,79	0,85	0,81	0,00	0,00	0,00	0,94	0,95	0,95
MedRoBERTa.nl	Sputum	800	0,58	0,62	0,60	0,78	0,90	0,84	0,00	0,00	0,00	0,96	0,95	0,95
MedRoBERTa.nl	Sputum	1000	0,80	0,70	0,71	0,80	0,89	0,84	0,64	0,25	0,33	0,96	0,95	0,95
MedRoBERTa.nl	Sputum	1200	0,83	0,77	0,79	0,84	0,87	0,86	0,68	0,49	0,56	0,95	0,95	0,95
MedRoBERTa.nl	Sputum	1400	0,83	0,79	0,80	0,82	0,88	0,85	0,71	0,56	0,61	0,96	0,94	0,95
MedRoBERTa.nl	Sputum	1600	0,81	0,78	0,78	0,86	0,87	0,86	0,63	0,51	0,54	0,95	0,95	0,95
RobBERT	Chest pain	200	0,28	0,33	0,31	0,00	0,00	0,00	0,00	0,00	0,00	0,85	1,00	0,92
RobBERT	Chest pain	400	0,28	0,33	0,31	0,00	0,00	0,00	0,00	0,00	0,00	0,85	1,00	0,92
RobBERT	Chest pain	600	0,56	0,51	0,49	0,54	0,58	0,53	0,20	0,01	0,03	0,92	0,94	0,93
RobBERT	Chest pain	800	0,57	0,57	0,54	0,61	0,57	0,52	0,16	0,20	0,16	0,93	0,94	0,94

						Per class metrics								
			Macro-averaged metrics			'Present'			'Absent'			'Not reported'		
**Model**	**Sign or symptom**	**Number of samples**	**Precision**	**Recall**	**F1-score**	**Precision**	**Recall**	**F1-score**	**Precision**	**Recall**	**F1-score**	**Precision**	**Recall**	**F1-score**
RobBERT	Chest pain	1000	0,66	0,63	0,61	0,59	0,68	0,62	0,46	0,29	0,29	0,94	0,93	0,94
RobBERT	Chest pain	1200	0,68	0,63	0,63	0,64	0,60	0,61	0,46	0,33	0,35	0,93	0,95	0,94
RobBERT	Chest pain	1400	0,72	0,60	0,60	0,58	0,70	0,62	0,65	0,16	0,24	0,94	0,95	0,94
RobBERT	Chest pain	1600	0,66	0,68	0,66	0,60	0,66	0,60	0,44	0,44	0,43	0,95	0,94	0,94
RobBERT	Chills	200	0,32	0,33	0,32	0,00	0,00	0,00	0,00	0,00	0,00	0,95	1,00	0,97
RobBERT	Chills	400	0,32	0,33	0,32	0,00	0,00	0,00	0,00	0,00	0,00	0,95	1,00	0,97
RobBERT	Chills	600	0,32	0,33	0,32	0,00	0,00	0,00	0,00	0,00	0,00	0,95	1,00	0,97
RobBERT	Chills	800	0,54	0,55	0,55	0,65	0,67	0,65	0,00	0,00	0,00	0,98	0,99	0,99
RobBERT	Chills	1000	0,56	0,59	0,57	0,70	0,77	0,73	0,00	0,00	0,00	0,99	1,00	0,99
RobBERT	Chills	1200	0,55	0,58	0,56	0,65	0,76	0,70	0,00	0,00	0,00	0,99	0,99	0,99
RobBERT	Chills	1400	0,63	0,65	0,64	0,88	0,95	0,91	0,00	0,00	0,00	1,00	1,00	1,00
RobBERT	Chills	1600	0,61	0,65	0,63	0,82	0,96	0,88	0,00	0,00	0,00	1,00	0,99	0,99
RobBERT	Confusion	200	0,32	0,33	0,33	0,00	0,00	0,00	0,00	0,00	0,00	0,97	1,00	0,98
RobBERT	Confusion	400	0,32	0,33	0,33	0,00	0,00	0,00	0,00	0,00	0,00	0,97	1,00	0,98
RobBERT	Confusion	600	0,32	0,33	0,33	0,00	0,00	0,00	0,00	0,00	0,00	0,97	1,00	0,98
RobBERT	Confusion	800	0,36	0,34	0,34	0,00	0,00	0,00	0,10	0,02	0,03	0,97	1,00	0,98
RobBERT	Confusion	1000	0,34	0,35	0,35	0,03	0,04	0,03	0,02	0,03	0,02	0,97	0,99	0,98
RobBERT	Confusion	1200	0,39	0,37	0,37	0,20	0,10	0,13	0,00	0,00	0,00	0,97	1,00	0,98
RobBERT	Confusion	1400	0,38	0,39	0,38	0,00	0,00	0,00	0,17	0,19	0,17	0,97	0,99	0,98
RobBERT	Confusion	1600	0,48	0,45	0,46	0,20	0,20	0,20	0,27	0,17	0,21	0,97	1,00	0,98
RobBERT	Cough	200	0,25	0,33	0,29	0,76	1,00	0,86	0,00	0,00	0,00	0,00	0,00	0,00
RobBERT	Cough	400	0,54	0,46	0,47	0,83	0,97	0,89	0,00	0,00	0,00	0,77	0,40	0,53
RobBERT	Cough	600	0,60	0,58	0,58	0,92	0,97	0,94	0,00	0,00	0,00	0,89	0,75	0,81
RobBERT	Cough	800	0,60	0,59	0,59	0,93	0,96	0,94	0,00	0,00	0,00	0,86	0,79	0,82
RobBERT	Cough	1000	0,61	0,60	0,60	0,94	0,97	0,95	0,00	0,00	0,00	0,88	0,83	0,85
RobBERT	Cough	1200	0,61	0,60	0,60	0,93	0,97	0,95	0,00	0,00	0,00	0,89	0,82	0,85
RobBERT	Cough	1400	0,62	0,60	0,61	0,94	0,98	0,96	0,00	0,00	0,00	0,91	0,83	0,87

						Per class metrics								
			Macro-averaged metrics			'Present'			'Absent'			'Not reported'		
Model	Sign or symptom	Number of samples	Precision	Recall	F1-score	Precision	Recall	F1-score	Precision	Recall	F1-score	Precision	Recall	F1-score
RobBERT	Cough	1600	0,61	0,58	0,59	0,92	0,98	0,95	0,00	0,00	0,00	0,90	0,77	0,82
RobBERT	Crackles upon auscultation	200	0,41	0,42	0,38	0,00	0,00	0,00	0,64	1,00	0,78	0,59	0,27	0,36
RobBERT	Crackles upon auscultation	400	0,87	0,71	0,76	0,89	0,56	0,67	0,79	0,97	0,87	0,93	0,61	0,73
RobBERT	Crackles upon auscultation	600	0,89	0,79	0,82	0,87	0,80	0,83	0,86	0,96	0,90	0,95	0,61	0,74
RobBERT	Crackles upon auscultation	800	0,88	0,81	0,84	0,85	0,85	0,84	0,88	0,94	0,91	0,91	0,65	0,76
RobBERT	Crackles upon auscultation	1000	0,87	0,83	0,85	0,84	0,88	0,86	0,90	0,93	0,92	0,88	0,68	0,76
RobBERT	Crackles upon auscultation	1200	0,88	0,82	0,84	0,88	0,85	0,86	0,89	0,94	0,91	0,86	0,68	0,74
RobBERT	Crackles upon auscultation	1400	0,85	0,85	0,85	0,83	0,91	0,87	0,92	0,91	0,91	0,80	0,73	0,76
RobBERT	Crackles upon auscultation	1600	0,89	0,84	0,86	0,85	0,88	0,86	0,91	0,93	0,92	0,90	0,72	0,79
RobBERT	Fever	200	0,28	0,36	0,26	0,07	0,00	0,00	0,34	0,18	0,20	0,44	0,89	0,59
RobBERT	Fever	400	0,54	0,46	0,42	0,48	0,08	0,13	0,41	0,84	0,55	0,72	0,48	0,57
RobBERT	Fever	600	0,63	0,58	0,58	0,65	0,42	0,49	0,47	0,69	0,56	0,76	0,62	0,68
RobBERT	Fever	800	0,69	0,69	0,68	0,61	0,80	0,69	0,70	0,49	0,57	0,77	0,79	0,78
RobBERT	Fever	1000	0,69	0,69	0,69	0,70	0,70	0,69	0,63	0,58	0,60	0,76	0,79	0,77
RobBERT	Fever	1200	0,72	0,69	0,68	0,73	0,66	0,66	0,63	0,64	0,61	0,80	0,77	0,77
RobBERT	Fever	1400	0,71	0,69	0,68	0,68	0,77	0,71	0,66	0,61	0,61	0,80	0,70	0,72
RobBERT	Fever	1600	0,71	0,71	0,70	0,69	0,78	0,73	0,66	0,58	0,61	0,78	0,76	0,77
RobBERT	Ill appearance	200	0,23	0,33	0,27	0,00	0,00	0,00	0,00	0,00	0,00	0,69	1,00	0,82
RobBERT	Ill appearance	400	0,74	0,64	0,61	0,63	0,10	0,16	0,68	0,86	0,76	0,90	0,94	0,92
RobBERT	Ill appearance	600	0,85	0,81	0,83	0,73	0,60	0,65	0,89	0,88	0,88	0,92	0,95	0,94
RobBERT	Ill appearance	800	0,88	0,82	0,84	0,82	0,59	0,68	0,89	0,89	0,89	0,93	0,96	0,94
RobBERT	Ill appearance	1000	0,85	0,85	0,85	0,72	0,72	0,71	0,88	0,89	0,89	0,94	0,93	0,94
RobBERT	Ill appearance	1200	0,89	0,84	0,85	0,81	0,65	0,72	0,91	0,89	0,90	0,93	0,96	0,95
RobBERT	Ill appearance	1400	0,86	0,84	0,84	0,74	0,67	0,69	0,90	0,88	0,89	0,94	0,95	0,94
RobBERT	Ill appearance	1600	0,88	0,83	0,84	0,79	0,62	0,68	0,90	0,90	0,90	0,93	0,96	0,94
RobBERT	Shortness of breath	200	0,39	0,41	0,36	0,46	0,85	0,59	0,00	0,00	0,00	0,71	0,37	0,47
RobBERT	Shortness of breath	400	0,43	0,46	0,42	0,51	0,87	0,64	0,00	0,00	0,00	0,78	0,52	0,62

						Per class metrics								
			Macro-averaged metrics			'Present'			'Absent'			'Not reported'		
**Model**	**Sign or symptom**	**Number of samples**	**Precision**	**Recall**	**F1-score**	**Precision**	**Recall**	**F1-score**	**Precision**	**Recall**	**F1-score**	**Precision**	**Recall**	**F1-score**
RobBERT	Shortness of breath	600	0,76	0,60	0,57	0,74	0,86	0,79	0,73	0,05	0,09	0,82	0,88	0,84
RobBERT	Shortness of breath	800	0,84	0,79	0,80	0,85	0,91	0,88	0,77	0,52	0,61	0,90	0,92	0,91
RobBERT	Shortness of breath	1000	0,84	0,80	0,82	0,85	0,91	0,88	0,77	0,59	0,67	0,91	0,91	0,91
RobBERT	Shortness of breath	1200	0,86	0,81	0,83	0,87	0,92	0,89	0,81	0,59	0,68	0,91	0,93	0,91
RobBERT	Shortness of breath	1400	0,87	0,81	0,83	0,85	0,94	0,89	0,83	0,58	0,68	0,92	0,91	0,91
RobBERT	Shortness of breath	1600	0,87	0,83	0,85	0,87	0,94	0,90	0,82	0,64	0,72	0,92	0,91	0,91
RobBERT	Sputum	200	0,24	0,33	0,28	0,00	0,00	0,00	0,00	0,00	0,00	0,73	1,00	0,85
RobBERT	Sputum	400	0,54	0,48	0,49	0,78	0,46	0,56	0,00	0,00	0,00	0,83	0,97	0,89
RobBERT	Sputum	600	0,58	0,60	0,59	0,80	0,83	0,81	0,00	0,00	0,00	0,94	0,96	0,95
RobBERT	Sputum	800	0,57	0,61	0,59	0,76	0,89	0,82	0,00	0,00	0,00	0,95	0,94	0,95
RobBERT	Sputum	1000	0,57	0,61	0,59	0,76	0,88	0,81	0,00	0,00	0,00	0,95	0,94	0,95
RobBERT	Sputum	1200	0,58	0,61	0,59	0,80	0,86	0,83	0,00	0,00	0,00	0,94	0,96	0,95
RobBERT	Sputum	1400	0,56	0,58	0,56	0,76	0,79	0,76	0,00	0,00	0,00	0,92	0,94	0,93
RobBERT	Sputum	1600	0,65	0,62	0,62	0,81	0,87	0,83	0,20	0,04	0,07	0,94	0,96	0,95

Table A4 | The performance of the MedRoBERTa.nl and RobBERT models as prompt-based classifier in terms of recall, precision, and F1-score for different sample sizes. The models were trained to extract a total of nine signs and symptoms related to lower respiratory tract Infection from Dutch primary care electronic health records.

						Per class metrics								
			Macro-averaged metrics			'Present'			'Absent'			'Not reported'		
**Model**	**Sign or symptom**	**Number of samples**	**Precision**	**Recall**	**F1-score**	**Precision**	**Recall**	**F1-score**	**Precision**	**Recall**	**F1-score**	**Precision**	**Recall**	**F1-score**
MedRoBERTa.nl	Chest pain	1	0,01	0,33	0,03	0,00	0,00	0,00	0,04	1,00	0,08	0,00	0,00	0,00
MedRoBERTa.nl	Chest pain	2	0,01	0,33	0,03	0,00	0,00	0,00	0,04	1,00	0,08	0,00	0,00	0,00
MedRoBERTa.nl	Chest pain	3	0,01	0,33	0,03	0,00	0,00	0,00	0,04	1,00	0,08	0,00	0,00	0,00
MedRoBERTa.nl	Chills	1	0,01	0,32	0,02	0,04	0,21	0,06	0,00	0,73	0,01	0,00	0,00	0,00
MedRoBERTa.nl	Chills	2	0,01	0,32	0,02	0,04	0,21	0,06	0,00	0,73	0,01	0,00	0,00	0,00
MedRoBERTa.nl	Chills	3	0,01	0,32	0,02	0,04	0,21	0,06	0,00	0,73	0,01	0,00	0,00	0,00
MedRoBERTa.nl	Confusion	1	0,34	0,33	0,05	0,01	0,92	0,02	0,00	0,02	0,00	1,00	0,06	0,11
MedRoBERTa.nl	Confusion	2	0,34	0,33	0,05	0,01	0,92	0,02	0,00	0,02	0,00	1,00	0,06	0,11
MedRoBERTa.nl	Confusion	3	0,34	0,33	0,05	0,01	0,92	0,02	0,00	0,02	0,00	1,00	0,06	0,11
MedRoBERTa.nl	Cough	1	0,33	0,33	0,02	0,73	0,01	0,02	0,01	0,97	0,03	0,25	0,01	0,02
MedRoBERTa.nl	Cough	2	0,33	0,33	0,02	0,73	0,01	0,02	0,01	0,97	0,03	0,25	0,01	0,02
MedRoBERTa.nl	Cough	3	0,33	0,33	0,02	0,73	0,01	0,02	0,01	0,97	0,03	0,25	0,01	0,02
MedRoBERTa.nl	Crackles upon auscultation	1	0,28	0,33	0,30	0,26	0,37	0,30	0,59	0,63	0,61	0,00	0,00	0,00
MedRoBERTa.nl	Crackles upon auscultation	2	0,28	0,33	0,30	0,26	0,37	0,30	0,59	0,63	0,61	0,00	0,00	0,00
MedRoBERTa.nl	Crackles upon auscultation	3	0,28	0,33	0,30	0,26	0,37	0,30	0,59	0,63	0,61	0,00	0,00	0,00
MedRoBERTa.nl	Fever	1	0,18	0,33	0,14	0,25	1,00	0,40	0,30	0,00	0,01	0,00	0,00	0,00
MedRoBERTa.nl	Fever	2	0,18	0,33	0,14	0,25	1,00	0,40	0,30	0,00	0,01	0,00	0,00	0,00
MedRoBERTa.nl	Fever	3	0,18	0,33	0,14	0,25	1,00	0,40	0,30	0,00	0,01	0,00	0,00	0,00
MedRoBERTa.nl	Ill appearance	1	0,18	0,34	0,07	0,11	1,00	0,20	0,44	0,01	0,03	0,00	0,00	0,00
MedRoBERTa.nl	Ill appearance	2	0,18	0,34	0,07	0,11	1,00	0,20	0,44	0,01	0,03	0,00	0,00	0,00
MedRoBERTa.nl	Ill appearance	3	0,15	0,34	0,07	0,11	1,00	0,20	0,34	0,01	0,03	0,00	0,00	0,00
MedRoBERTa.nl	Shortness of breath	1	0,04	0,33	0,07	0,00	0,00	0,00	0,12	1,00	0,22	0,00	0,00	0,00
MedRoBERTa.nl	Shortness of breath	2	0,04	0,33	0,07	0,00	0,00	0,00	0,12	1,00	0,22	0,00	0,00	0,00
MedRoBERTa.nl	Shortness of breath	3	0,04	0,33	0,07	0,00	0,00	0,00	0,12	1,00	0,22	0,00	0,00	0,00
MedRoBERTa.nl	Sputum	1	0,01	0,33	0,02	0,00	0,00	0,00	0,03	1,00	0,05	0,00	0,00	0,00

			Macro-averaged metrics			Per class metrics								
						'Present'			'Absent'			'Not reported'		
**Model**	**Sign or symptom**	**Number of samples**	**Precision**	**Recall**	**F1-score**	**Precision**	**Recall**	**F1-score**	**Precision**	**Recall**	**F1-score**	**Precision**	**Recall**	**F1-score**
MedRoBERTa.nl	Sputum	2	0,01	0,33	0,02	0,00	0,00	0,00	0,03	1,00	0,05	0,00	0,00	0,00
MedRoBERTa.nl	Sputum	3	0,01	0,33	0,02	0,00	0,00	0,00	0,03	1,00	0,05	0,00	0,00	0,00
RobBERT	Chest pain	1	0,01	0,33	0,03	0,00	0,00	0,00	0,04	1,00	0,08	0,00	0,00	0,00
RobBERT	Chest pain	2	0,01	0,33	0,03	0,00	0,00	0,00	0,04	1,00	0,08	0,00	0,00	0,00
RobBERT	Chest pain	3	0,01	0,33	0,03	0,00	0,00	0,00	0,04	1,00	0,08	0,00	0,00	0,00
RobBERT	Chills	1	0,02	0,33	0,03	0,05	0,99	0,09	0,00	0,00	0,00	0,00	0,00	0,00
RobBERT	Chills	2	0,02	0,33	0,03	0,05	0,99	0,09	0,00	0,00	0,00	0,00	0,00	0,00
RobBERT	Chills	3	0,02	0,33	0,03	0,05	0,99	0,09	0,00	0,00	0,00	0,00	0,00	0,00
RobBERT	Confusion	1	0,01	0,31	0,02	0,02	0,12	0,03	0,02	0,80	0,04	0,00	0,00	0,00
RobBERT	Confusion	2	0,01	0,31	0,02	0,02	0,12	0,03	0,02	0,80	0,04	0,00	0,00	0,00
RobBERT	Confusion	3	0,01	0,31	0,02	0,02	0,12	0,03	0,02	0,80	0,04	0,00	0,00	0,00
RobBERT	Cough	1	0,07	0,32	0,02	0,00	0,00	0,00	0,01	0,95	0,03	0,19	0,02	0,03
RobBERT	Cough	2	0,07	0,32	0,02	0,00	0,00	0,00	0,01	0,95	0,03	0,21	0,02	0,03
RobBERT	Cough	3	0,07	0,32	0,02	0,00	0,00	0,00	0,01	0,95	0,03	0,21	0,02	0,03
RobBERT	Crackles upon auscultation	1	0,08	0,33	0,13	0,23	1,00	0,38	0,00	0,00	0,00	0,00	0,00	0,00
RobBERT	Crackles upon auscultation	2	0,08	0,33	0,13	0,23	1,00	0,38	0,00	0,00	0,00	0,00	0,00	0,00
RobBERT	Crackles upon auscultation	3	0,08	0,33	0,13	0,23	1,00	0,38	0,00	0,00	0,00	0,00	0,00	0,00
RobBERT	Fever	1	0,20	0,33	0,22	0,24	0,75	0,37	0,37	0,25	0,30	0,00	0,00	0,00
RobBERT	Fever	2	0,20	0,33	0,22	0,24	0,75	0,37	0,37	0,25	0,30	0,00	0,00	0,00
RobBERT	Fever	3	0,21	0,33	0,22	0,24	0,75	0,37	0,37	0,25	0,30	0,00	0,00	0,00
RobBERT	Ill appearance	1	0,04	0,33	0,06	0,11	1,00	0,19	0,00	0,00	0,00	0,00	0,00	0,00
RobBERT	Ill appearance	2	0,04	0,33	0,06	0,11	1,00	0,19	0,00	0,00	0,00	0,00	0,00	0,00
RobBERT	Ill appearance	3	0,04	0,33	0,06	0,11	1,00	0,19	0,00	0,00	0,00	0,00	0,00	0,00
RobBERT	Shortness of breath	1	0,04	0,33	0,07	0,00	0,00	0,00	0,12	1,00	0,22	0,00	0,00	0,00
RobBERT	Shortness of breath	2	0,04	0,33	0,07	0,00	0,00	0,00	0,12	1,00	0,22	0,00	0,00	0,00
RobBERT	Shortness of breath	3	0,04	0,33	0,07	0,00	0,00	0,00	0,12	1,00	0,22	0,00	0,00	0,00
RobBERT	Sputum	1	0,08	0,33	0,13	0,24	1,00	0,38	0,00	0,00	0,00	0,00	0,00	0,00

						Per class metrics								
			Macro-averaged metrics			'Present'			'Absent'			'Not reported'		
**Model**	**Sign or symptom**	**Number of samples**	**Precision**	**Recall**	**F1-score**	**Precision**	**Recall**	**F1-score**	**Precision**	**Recall**	**F1-score**	**Precision**	**Recall**	**F1-score**
RobBERT	Sputum	2	0,08	0,33	0,13	0,24	1,00	0,38	0,00	0,00	0,00	0,00	0,00	0,00
RobBERT	Sputum	3	0,08	0,33	0,13	0,24	1,00	0,38	0,00	0,00	0,00	0,00	0,00	0,00

TRIPOD+LLM Checklist

Section / Topic	Item Number	Checklist Item	Research Design	LLM Task	Reported on Page
**Abstract**					
**Title**	2a	Identify the study as developing, fine-tuning, and/or evaluating the performance of an LLM, specifying the task, the target population, and the outcome to be predicted.	All	All	1
**Abstract**	2b	Provide a brief explanation of the healthcare context, use case and rationale for developing or evaluating the performance of an LLM.	E,H	All	2
**Objectives**	2c	Specify the study objectives, including whether the study describes LLMs development, tuning, and/or evaluation	All	All	3,4
**Methods**	2d	Describe the key elements of the study setting.	All	All	5, 6
	2e	Detail all data used in the study, specify data splits and any selective use of data.	M,D,E	All	5
	2f	Specify the name and version of LLM used.	All	All	7, 8, 9
	2g	Briefly summarize the LLM-building steps, including any fine-tuning, reward modeling, reinforcement learning with human feedback (RLHF), etc.	M,D	All	7, 8, 9, 10, appendix
	2h	Describe the specific tasks performed by the LLMs (e.g., medical QA, summarization, extraction), highlighting key inputs and outputs used in the final LLM.	All	All	6
	2i	Specify the evaluation datasets/populations used, including the endpoint evaluated, and detail whether this information was held out during training/tuning where relevant, and what measure(s) were used to evaluate LLM performance.	All	All	5, 6, 11, 12
**Results**	2j	Give an overall report and interpretation of the main results.	All	All	11-14
**Discussion**	2k	Explicitly state any broader implications or concerns that have arisen in light of these results.	All	All	15, 16, 17
**Other**	2l	Give the registration number and name of the registry or repository (if relevant).	H	All	Not Required
**Introduction**					

Section / Topic	Item Number	Checklist Item	Research Design	LLM Task	Reported on Page
**Background**	3a	Explain the healthcare context / use case (e.g., administrative, diagnostic, therapeutic, clinical workflow) and rationale for developing or evaluating the LLM, including references to existing approaches and models.	All	All	5
	3b	Describe the target population and the intended use of the LLM in the context of the care pathway, including its intended users in current gold standard practices (e.g., healthcare professionals, patients, public, or administrators).	E,H	All	1-5
**Objectives**	4	Specify the study objectives, including whether the study describes the initial development, fine-tuning, or validation of an LLM (or multiple stages).	All	All	4
**Methods**					
**Data**	5a	Describe the sources of data separately for the training, tuning, and/or evaluation datasets and the rationale for using these data (e.g., web corpora, clinical research/trial data, EHR data).	All	All	5, 9
	5b	Describe the relevant data points and provide a quantitative and qualitative description of their distribution and other relevant descriptors of the dataset (e.g., source, languages, countries of origin)	All	All	5, 6
	5c	Specifically state the date of the oldest and newest item of text used in the development process (training, fine-tuning, reward modeling) and in the evaluation datasets.	M,D,E,H	All	5
	5d	Describe any data pre-processing and quality checking, including whether this was similar across text corpora, institutions, and relevant sociodemographic groups.	All	All	5,6
	5e	Describe how missing and imbalanced data were handled and provide reasons for omitting any data.	M,D,E	All	6

Section / Topic	Item Number	Checklist Item	Research Design	LLM Task	Reported on Page
Analytical Methods	6a	Report the LLM name, version, and last date of training or use during inference.	All	All	8,9
	6b	Specify the type of LLM architecture, and LLM building steps, including any hyperparameter tuning (e.g., temperature, length limits, penalties), prompt engineering, and any inference settings (e.g., seed, temperature, max token length) as relevant.	M,D,E	All	8,9
	6c	Report details of LLM development process from text input to outcome generation, such as training, fine-tuning procedures, and alignment strategy (e.g., reinforcement learning, direct preference optimization, etc.) and alignment goals (e.g., helpfulness, honesty, harmlessness, etc.).	M,D	All	9
	6d	Specify the initial and post-processed output of the LLM (e.g., probabilities, classification, unstructured text).	All	All	7-9
	6e	Provide details and rationale for any classification and how the probabilities were determined and thresholds identified.	All	C,OF	Not Required
	6f	Include metrics that capture the quality of generative outputs, such as consistency, relevance, and accuracy, compared to gold standards.	All	QA,IR,DG,SS,MT	Not Required
	6g	Report the outcome metrics' relevance to downstream task at deployment time and correlation of metric to human evaluation of the text for the intended use.	E,H	All	10,11
LLM Output	7a	Clearly define the outcome, how the LLM predictions were calculated (e.g., formula, code, object, API), and evaluation metrics.	E,H	All	10,11
	7b	If outcome assessment requires subjective interpretation, describe the qualifications of the assessors, any instructions provided, relevant information on demographics of the assessors, and inter-assessor agreement.	All	All	6
	7c	Specify how performance was compared to other LLMs, humans, and other benchmarks or standards.	All	All	11,12,13, appendix

Section / Topic	Item Number	Checklist Item	Research Design	LLM Task	Reported on Page
**Annotation**	8a	If annotation was done, report how text was labeled, including providing specific annotation guidelines with examples.	All	All	6
	8b	If annotation was done, report how many annotators labeled the dataset(s), including the proportion of data in each dataset that were annotated by more than 1 annotator.	All	All	6
	8c	If annotation was done, provide information on the background and experience of the annotators, and the inter-annotator agreement.	All	All	6
**Prompting**	9a	If research involved prompting LLMs, provide details on the processes used during prompt design, curation, and selection.	All	All	9,10
	9b	If research involved prompting LLMs, report what data were used to develop the prompts.	All	All	9
**Summarization**	10	Describe any preprocessing of the data before summarization.	All	SS	Not Required
**Instruction Tuning / Alignment**	11	If instruction tuning/alignment strategies were used, what were the instructions and interface used for evaluation, and what were the characteristics of the populations doing evaluation?	M,D	All	N/A
**Compute**	12	Report compute, or proxies thereof (e.g., time on what and how many machines, cost on what and how many machines, inference time, floating-point operations per second (FLOPs)), required to carry out methods.	M,D,E	All	11
**Ethics Approval**	13	Name the institutional research board or ethics committee that approved the study and describe the participant-informed consent or the ethics committee waiver of informed consent.	All	All	11

Section / Topic	Item Number	Checklist Item	Research Design	LLM Task	Reported on Page
**Open Science**	14a	Give the source of funding and the role of the funders for the present study.	All	All	17,18
	14b	Declare any conflicts of interest and financial disclosures for all authors.	All	All	N/A
	14c	Indicate where the study protocol can be accessed or state that a protocol was not prepared.	H	All	Not Required
	14d	Provide registration information for the study, including register name and registration number, or state that the study was not registered.	H	All	Not Required
	14e	Provide details of the availability of the study data.	All	All	18
	14f	Provide details of the availability of the code to reproduce the study results.	All	All	18
**Public Involvement**	15	Provide details of any patient and public involvement during the design, conduct, reporting, interpretation, or dissemination of the study or state no involvement.	H	All	Not Required
**Results**					
**Participants**	16a	When using patient/EHR data, describe the flow of text/EHR/patient data through the study, including the number of documents/questions/participants with and without the outcome/label and follow-up time.	E,H	All	5
	16b	When using patient/EHR data, report the characteristics overall and, for each data source or setting, and for development/evaluation splits, including the key dates, key predictors, and sample size.	E,H	All	5, 6
	16c	For LLM evaluation, show a comparison of the distribution of important predictors between development and evaluation data.	E,H	All	6
	16d	When using patient/EHR data, specify the number of participants and outcome events in each analysis (e.g., for LLM development, hyperparameter tuning, LLM evaluation).	E,H	All	5,6
**Performance**	17	Report LLM performance according to pre-specified metrics (see item 7a) and/or human evaluation (see item 7d).	All	All	11-14, appendix
**LLM Updating**	18	If applicable, report the results from any LLM updating, including the updated LLM and subsequent performance.	All	All	N/A
**Discussion**					

Section / Topic	Item Number	Checklist Item	Research Design	LLM Task	Reported on Page
**Interpretation**	19a	Give an overall interpretation of the main results, including issues of fairness in the context of the objectives and previous studies.	All	All	15-17
**Limitations**	19b	Discuss any limitations of the study and their effects on any biases, statistical uncertainty, and generalizability.	All	All	16,17
**Usability of the LLM in context**	19c	Describe any known challenges in using data for the specified task and domain context with reference to representation, missingness, harmonization, and bias.	E,H	All	15-17
	19d	Define the intended use for the implementation under evaluation, including the intended input, end-user, level of autonomy/human oversight.	E,H	All	17
	19e	If applicable, describe how poor quality or unavailable input data should be assessed and handled when implementing the LLM, i.e., what is the usability of the LLM in the context of current clinical care.	E,H	All	N/A
	19f	If applicable, specify whether users will be required to interact in the handling of the input data or use of the LLM, and what level of expertise is required of users.	E,H	All	N/A
	19g	Discuss any next steps for future research, with a specific view to applicability and generalizability of the LLM.	All	All	17
